# Neuropharmacological characterization of the oneirogenic Mexican plant *Calea zacatechichi* aqueous extract in mice

**DOI:** 10.1007/s11011-016-9794-1

**Published:** 2016-01-28

**Authors:** Maciej Sałaga, Jakub Fichna, Katarzyna Socała, Dorota Nieoczym, Mateusz Pieróg, Marta Zielińska, Anna Kowalczuk, Piotr Wlaź

**Affiliations:** Department of Biochemistry, Faculty of Medicine, Medical University of Lodz, Łódź, Poland; Department of Animal Physiology, Institute of Biology and Biochemistry, Maria Curie-Sklodowska University, Akademicka 19, 20-033 Lublin, PL Poland; National Medicines Institute, Warsaw, Poland

**Keywords:** Anticonvulsant activity, Seizure threshold, Abdominal pain, Mice

## Abstract

This study evaluates the neuropharmacological effects of the aqueous extract of the Mexican plant *Calea zacatechichi* Schltdl., which is commonly used in folk medicine to treat cough, asthma, and gastrointestinal disorders. Moreover, it has been used for centuries in traditional rituals based on divination and is thought to possess hallucinogenic activity. To test the neuropharmacological effects of the aqueous extract of *C. zacatechichi* we used mouse models of convulsions, an elevated plus-maze test and measured locomotor activity. We also evaluated the effect of the extract on antidepressant-like behavior in forced swim test, as well as on muscular strength in a grip test. Moreover the antinociceptive action of the extract was evaluated in the hot-plate and writhing tests. The chemical composition of the extract was evaluated using LC-MS techniques. The aqueous extract of *C. zacatechichi* did not affect any of the parameters measured in seizure models. It had also no influence on anxiety, exploratory behavior and muscular strength in the applied doses. On the other hand, the extract exhibited antinociceptive effect in the mouse model of abdominal pain. Chemical characterization of the extract showed the presence of chlorogenic acid, acacetin, and germacranolides. Based on this report we suggest that aqueous extract of *C. zacatechichi* has insignificant neuropharmacological effects in vivo and reduces abdominal pain perception. Our results, together with previous studies showing beneficial effects of the extracts obtained from *C. zacatechichi* suggest that these preparations may be used to treat medical conditions.

## Introduction

*Calea zacatechichi* Schltdl. (*Asteraceae* alt. *Compositae*) is a Latin-American plant, also known as Dream Herb or Bitter Grass, which grows predominantly in south-eastern Mexico. Native Indian tribes have used it for centuries as a remedy for cough and asthma, as well as gastrointestinal (GI) tract disorders, such as stomach-ache and diarrhea (Leonti et al. 2003). To date, several scientific reports have shown beneficial effects of *C. zacatechichi* extracts. For example, Wu et al. (2011) characterized anti-microbial and anti-leishmanial activity of compounds isolated from *C. zacatechichi*. Venegas-Flores et al. (2002) reported anti-inflammatory properties of *C. zacatechichi* aqueous extract. Furthermore, Bork et al. (1997) demonstrated that *C. zacatechichi* leaves extract negatively interfered with activation of Nf-κB transcriptional factor. Most recently, Sałaga et al. (2015) showed that dichloromethane extract of *C. zacatechichi* has anti-diarrheal and anti-nociceptive effects in the GI tract.

Apart from its medicinal properties, *C. zacatechichi* is also known to have some central nervous system (CNS)-related effects. Several Indian tribes, such as Chontal Indians used *C. zacatechichi* for rituals aimed at dream-based divination (Wu et al. 2011). In line, it has been reported that *C. zacatechichi* has some hallucinogenic properties and may affect sleep. Its organic extracts induce somnolence-like behaviors accompanied by the changes in EEG and light sleep in cats (Mayagoitia et al. 1986). Large doses elicit salivation, ataxia and retching (Mayagoitia et al. 1986). A double-blind human study by Mayagoitia et al. demonstrated that in healthy volunteers, low doses of *C. zacatechichi* extracts increased reaction time and time-lapse estimation (Mayagoitia et al. 1986). Moreover, it has been shown that the effects of the plant upon cingulum discharge frequency were significantly different from other hallucinogenic drugs, such as ketamine, quipazine and phencyclidine (Mayagoitia et al. 1986). In the light of the currently available scientific literature it is difficult to estimate whether therapeutic effects of *C. zacatechichi* extracts would be affected by CNS-related effects and, if any, which types of extracts are particularly abundant in hallucinogenic compounds. To shed light on the neuropsychopharmacological profile of the plant and its constituents, we investigated the effect of *C. zacatechichi* aqueous extract on seizure threshold, muscular strength, mood-related symptoms and locomotor activity in mice. Furthermore we assessed the antinociceptive properties of the extract in models of centrally- and peripherally-mediated antinociception. We also characterized the chemical composition of the extract by mass spectrometry to find novel biologically active compounds responsible for its potential effects.

## Materials and methods

### Plant material

Dried shredded herb of *Calea zacatechichi* (leaves, stems and flowers) was purchased from the company Maya Ethnobotanicals (Haarlem, Netherlands). The material originated from Mexico according to the provider declaration. The authenticity of the purchased material was confirmed through the macroscopic and microscopic assessment which was carried out in comparison to the authenticated *C. zacatechichi* material from Daniel Siebert, provided by The University of Mississippi School of Pharmacy, Mississippi, MS, USA. The plant name has been checked with http://www.theplantlist.org on 30–10-2013.

### Extract preparation

Powdered *C. zacatechichi* herb (150 g) was extracted 4 times with 2 l of boiling water. The aqueous solution was filtered and lyophilized. *C. zacatechichi* aqueous extract was prepared in the National Medicines Institute, Warsaw, Poland.

### Animals

Experimentally naïve, age matched male albino Swiss mice (Laboratory Animals Breeding, Słaboszów, Poland) weighing 22–30 g were used in all experiments. The animals were housed in Makrolon cages under strictly controlled laboratory conditions (22–23 °C, relative humidity, 45–55 %, 12 h light/dark cycle, lights on at 6:00 a.m.). Chow pellets (Agropol S.J., Motycz, Poland) and tap water were available ad libitum*.* After at least 7 days of acclimatization, experiments were performed between 8:00 and 16:00 h to minimize circadian influences. Each animal was used only once. The experimental protocol was in accordance to the European Communities Council Directive of 22 September 2010 (2010/63/EU) and Polish legislation acts concerning animal experimentations and was approved by the Local Ethics Committee at the Medical University of Lublin (license number 45/2013).

### Drug administration

All drugs and reagents, unless stated otherwise, were purchased from Sigma-Aldrich (Poznań, Poland). The *C. zacatechichi* aqueous extract (powder) was diluted in saline to desired concentrations and administered orally (p.o.) by gastric gavage in a volume of 10 ml/kg 60 min before the respective test. In time course experiments the *C. zacatechichi* extract was administered p.o. 15, 30, 60, 120 and 240 min before the test. Control animals received saline alone (p.o.). The vehicles in the used concentrations had no effects on the observed parameters. The dilutions of the *C. zacatechichi* extract used in this study were chosen based on preliminary experiments, in reference to doses used in humans.

### Maximal electroshock seizure threshold (MEST) test in mice

The electroshock seizures were induced by sine-wave alternating current (maximal output voltage 500 V, 50 Hz for 0.2 s) applied via transcorneal electrodes. The current was delivered by a rodent shocker (type 221; Hugo Sachs Elektronik, Freiburg, Germany). An ocular anesthetic (1 % solution of tetracaine hydrochloride) was applied into each eye 1 min before stimulation to minimize the pain. Transcorneal electrodes were soaked in 0.9 % saline to maximize the conductance. During stimulation mice were manually immobilized and immediately after the stimulation they were placed in a Plexiglas arena (37 cm × 21 cm × 14 cm) for behavioral observation. The presence or absence of seizure activity was recorded. Tonic hindlimb extension was taken as an endpoint.

The thresholds for maximal electroconvulsions were assessed by the ‘up-and-down’ method described by Kimball et al. (1957). In this method, current intensity was lowered or raised by 0.06-log intervals depending on whether the previously stimulated animal did or did not exert seizure activity, respectively. Each mouse was stimulated only once at any given current intensity. Experimental groups comprised 18–20 animals. Obtained data was used to determine the threshold current causing an endpoint in 50 % of mice (CC_50_ with confidence limits for 95 % probability). For further methodological detail see: Giardina and Gasior (2009).

### The 6 Hz psychomotor seizure threshold test in mice

The psychomotor seizure thresholds were examined using square-wave alternating current stimuli (0.2-ms duration pulses at 6 Hz for 3 s) delivered via saline-soaked corneal electrodes by a Grass S48 stimulator coupled with a constant current unit CCU1 (both from Grass Technologies, West Warwick, RI, USA). Ocular anesthesia and observation arena were made as described in above section. The seizures induced by 6 Hz stimulation were characterized by immobility or stun posture, which was frequently followed by rearing, forelimb clonus, twitching of the vibrissae and elevated or Straub tail (Barton et al. 2001; Barton et al. 2003). Renewal of normal exploratory behavior or the absence of the features listed above within 10 s after stimulation were considered as the lack of seizures. The ‘up-and-down’ method described by Kimball et al. (1957) was used in order to choose the current intensity. Data obtained in groups of 19–20 animals were used to determine the threshold current causing 6 Hz-induced seizures in 50 % of mice (CC_50_ with confidence limits for 95 % probability).

### The intravenous pentylenetetrazole (PTZ) seizure threshold test in mice

Briefly, mice were placed in a cylindrical plastic restrainer (12-cm long, 3-cm inner diameter) and a 27-gauge needle (Sterican®, B. Braun Melsungen, Melsungen, Germany) was inserted into the lateral tail vein. The needle was attached by polyethylene tubing (PE20RW, Plastics One Inc., Roanoke, VA, USA) to a plastic syringe, which was mounted in a syringe pump (model Physio 22, Hugo Sachs Elektronik–Harvard Apparatus GmbH, March-Hugstetten, Germany). The correct needle placement in the tail vein was verified by the appearance of blood in the tubing. The 1 % aqueous solution of PTZ, was administered into the lateral tail vein of unrestrained animals at a constant rate of 0.2 ml/min. Endpoints recorded to determine seizure thresholds: (1) the initial myoclonic twitch, (2) the onset generalized clonus with loss of righting reflex and (3) the onset of tonic forelimb extension. The time elapsed from the start of PTZ infusion to the onset of all three seizure stages was measured. The thresholds were calculated separately for each endpoint according to the formula: threshold dose of PTZ (mg/kg) = (infusion duration (s) × infusion rate (ml/s) × PTZ concentration (mg/ml) × 1000)/body weight (kg). Seizure thresholds were expressed as the amount of PTZ (mg/kg) ± SEM needed to produce the first sign of each endpoint in groups of 10–14 mice.

### Forced swim test

Forced swim test was performed according to the modified method of Porsolt et al. (1977). Mice (10–12/group) were individually placed into the glass cylinders (height 25 cm, diameter 10 cm) containing 10 cm of water maintained at 23–25 °C and left in it for 6 min. The total duration of immobility was recorded during the last 4 min of the 6-min testing period. The mice were considered immobile when they remained floating motionless in the water, making only the slow movements necessary to keep their heads above the water.

### Elevated plus-maze test

To identify anxiolytic properties of the studied extract we used elevated plus-maze test in mice, as described earlier by Lister (1987). The plus-maze apparatus was made of black polyvinyl chloride and consisted of two open (30 × 5 cm) and two enclosed (30 × 5 × 15 cm) arms. The arms extended from a central platform of 5 × 5 cm. The apparatus was mounted 38 cm above the floor level and was illuminated by red light. Animals (12 mice per group) were placed in the center of the apparatus (facing an enclosed arm), allowed to freely explore the maze and observed for 5 min. The following parameters were recorded: total number of arm entries, number of entries into the open arms and the time spent in these arms. An entry was defined as crossing the boundaries of the arms with all four paws. The percentage of entries and time spent in the open arms were calculated.

### The grip-strength test in mice

The effect of the studied extract on skeletal muscle strength in mice was measured in the grip-strength test, as described before (Nieoczym et al. 2010). The grip-strength apparatus (BioSeb, Chaville, France) consisted of a steel wire grid (8 × 8 cm) connected to an isometric force transducer. Animals (12 mice/group) were lifted by the tail so that they could grasp the grid with their forepaws. Then animals were pulled backward gently by the tail until they released the grid. The maximal grip strength value (in newtons, N) of the animal was recorded. The procedure was repeated three times and the mean force exerted by each mouse was recorded. The mean force was normalized to body weight and expressed in N/g.

### Locomotor activity

To monitor the spontaneous locomotor activity of mice, an IR Actimeter system (Panlab/Harvard Apparatus, Barcelona, Spain) was used. This consisted of a square arena surrounded by a 25 × 25 cm frame, containing a total of 16 × 16 infrared beams located on the sides and spaced at 1.5 cm intervals. Mice (11 per group) pretreated with *C. zacatechichi* aqueous extract or vehicle were placed individually in the actimeter and allowed to freely explore the arena for 6 min. The number of crosses of the infrared beams was counted by the device within last 4 min of the test, what corresponds to the time interval analyzed in the forced swim test.

### Behavioral pain responses

#### Hot-plate test

The hot-plate test was performed according to the method described earlier (Eddy and Leimbach 1953; Sobczak et al. 2014). A transparent plastic cylinder (14 cm diameter, 20 cm height) was used to confine the mouse on the heated (55 ± 0.5 °C) metal plate. Animals (12 mice/group) were administered with vehicle or *C. zacatechichi* aqueous extract (200 mg/kg, p.o.) and gently placed on the hot-plate. The latencies to paw licking, rearing and jumping were measured. A cut-off time of 240 s was used to avoid thermal skin injury. The dose of 200 mg/kg used in behavioral pain tests was selected, as the lowest from the doses tested in neuropharmacological tests which did not cause any CNS-related adverse events.

#### Writhing test

The writhing test was performed as described earlier (Laird et al. 2001; Sałaga et al. 2014). After administration of vehicle or *C. zacatechichi* aqueous extract (200 mg/kg, p.o.), mice (12 mice/group) were injected intraperitoneally (i.p.) with 0.75 % (*v*/v in saline) acetic acid solution (10 ml/kg). After 5 min of recovery, mice were placed in individual observational cages and the total number of writhes was counted during three periods of 5 min each. The writhing response, regarded as a nociceptive behavior, was characterized by elongation of the body and the development of tension in the abdominal muscles and hind paws.

### LC-MS method

A tandem mass spectrometer MicrOTOF-Q II from Bruker Daltonics (Bremen, Germany) coupled with an LC-UV Ultimate 3000 system (Dionex, a part of Thermo Fisher Scientific) was used to obtain the electrospray ionization time-of-flight mass spectra (LC-ESI-MS/MS-TOF). The following settings were used: electrospray ionization (ESI) in the positive ion mode, dry gas (nitrogen) flow rate 8.0 l/min, the dry heater 180 °C, the capillary voltage 4500 V and end plate offset −500 V. MS data were recorded in the full scan mode (from 50 to 3000 m/z). Data processing was carried out with Compass 1.3 (Bruker Daltonics).

Chromatographic analysis was performed on a C_18_ analytical column (Hypersil GOLD, 150 mm × 2.1 mm; 3 μm particle size; Thermo Fisher Scientific, Waltham, MA, USA) with a guard column (Hypersil GOLD, 10 mm × 2.1 mm; 3 μm particle size; Thermo Fisher Scientific, Walthman, MA, USA). The linear gradient elution was performed using 0.1 % formic acid in solvent A (water/acetonitrile, 9:1, v:v) and 0.1 % formic acid in solvent B (methanol/acetonitrile, 9:1, v:v). An applied linear gradient was as follows: initially 10 % B from 0 to 2 min, then linear gradient to 90 % B at 7 min, constant 90 % B to 10 min, finally return to 10 % B and equilibration for 2 min. The flow rate was 0.15 ml/min and the injection volume was 2 μl. The analysis was carried out at 25 °C.

To identify individual compounds we analyzed the high resolution mass spectra. TOF analyzer enables a very accurate mass detection and the assignment to the most probable molecular formula. Suggested molecular formulas were accepted when the mass error was below 5 ppm. Additionally we enhanced the identification by the analysis of the isotopic pattern of the compounds. When possible, obtained MS/MS spectra were additionally compared with spectra of identified compounds in the METLIN: Metabolite and Tandem MS Database (http://metlin.scripps.edu).

### Statistical analysis

Data obtained from the MEST test and the 6 Hz seizure threshold test were analyzed according to Kimball et al. (1957) and are presented as CC_50_ values with 95 % confidence limits. Differences in CC_50_ values were analyzed by using one-way analysis of variance (ANOVA) followed by Dunnett’s post hoc test. All other results are expressed as means ± SEM and were analyzed by one-way ANOVA followed by Dunnett’s post hoc test or Student’s *t* test (locomotor activity). Statistical significance was noted when *p* values were equal to or less than 0.05. All statistical calculations were performed with GraphPad Prism version 5.03 for Windows (GraphPad Software, San Diego, CA, USA).

## Results

### Aqueous extract of *C. zacatechichi* does not affect the threshold in the MEST test in mice

**Fig. 1:**
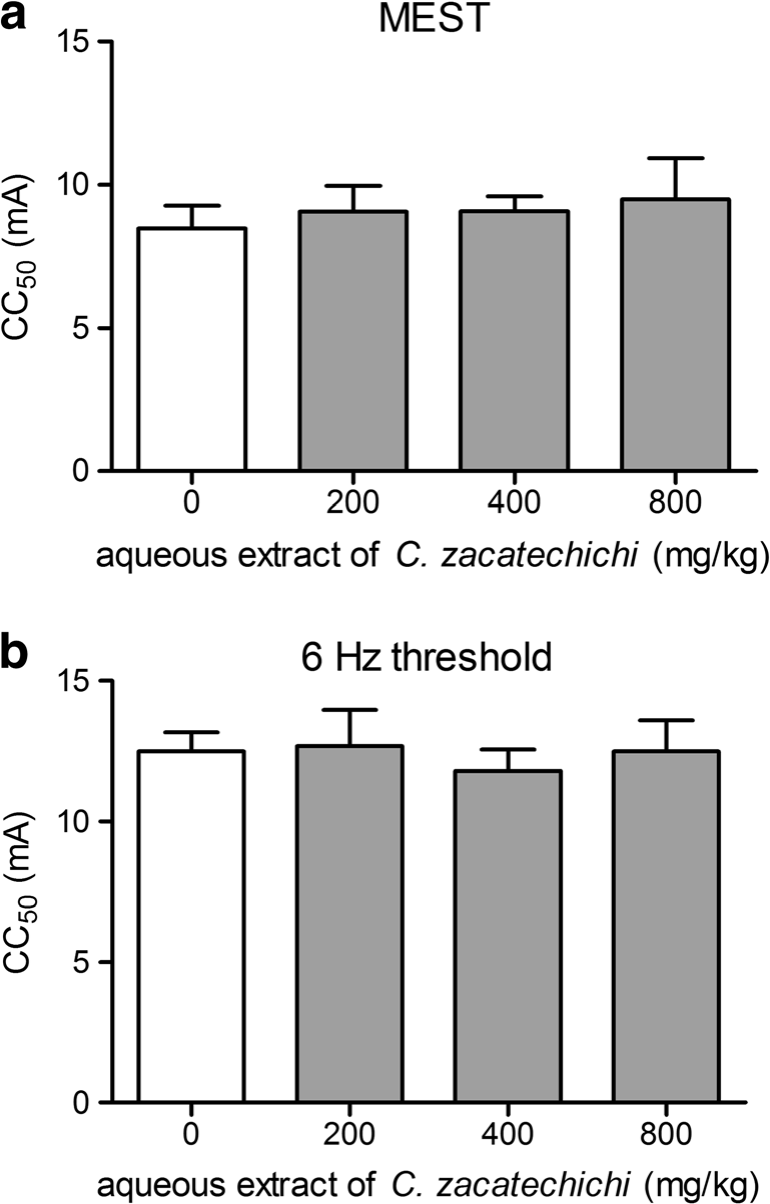
Effect of the aqueous extract of *C. zacatechichi* (200–800 mg/kg, p.o.) on maximal electroshock seizure threshold and 6 Hz psychomotor seizure threshold in mice. Figure shows data for CC_50_ of the MEST (**a**) and 6 Hz seizure threshold (**b**). Data represent mean + SEM of *n* = 18–20 mice for each experimental group

To establish whether aqueous extract of *C. zacatechichi* has the ability to prevent seizure spread through neural tissue, we used MEST test. Aqueous extract of *C. zacatechichi* administered at doses ranging from 200 to 800 mg/kg (p.o.) did not affect the MEST (Fig. 1a).

### Aqueous extract of *C. zacatechichi* does not influence the 6 Hz psychomotor seizure threshold in mice

To evaluate the potential influence of aqueous extract of *C. zacatechichi* on partial psychomotor seizures, we used 6 Hz psychomotor seizure threshold test. As shown in Fig. 1b the aqueous extract of *C. zacatechichi* administered at doses ranging from 200 to 800 mg/kg (p.o.) did not affect the 6 Hz seizure threshold.

### Aqueous extract of *C. zacatechichi* does not affect PTZ seizure threshold in mice

**Fig. 2:**
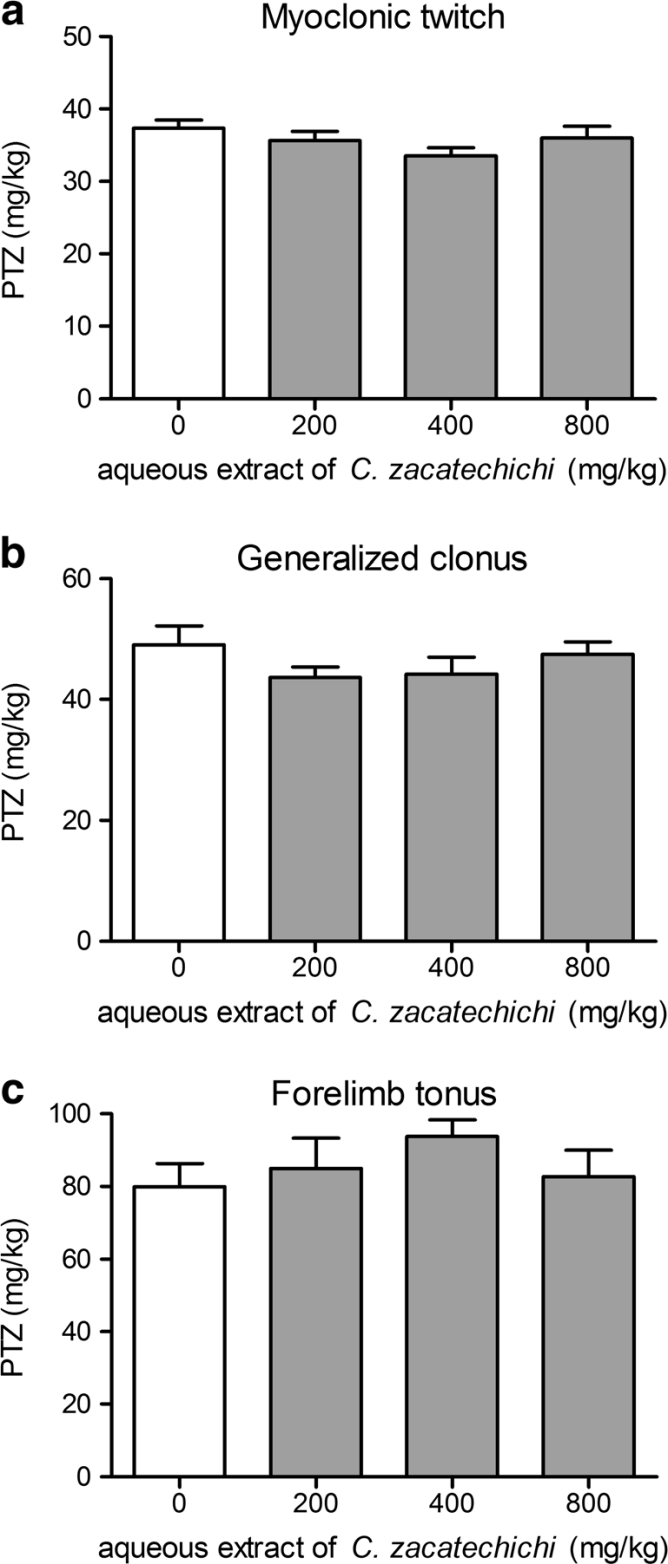
Effect of the aqueous extract of *C. zacatechichi* (200–800 mg/kg, p.o.) on the pentylenetetrazole seizure threshold in mice. Figure shows data for myoclonic twitch (**a**), generalized clonus (**b**) and forelimb tonus (**c**). Data represent mean + SEM of *n* = 10–13 mice for each experimental group

**Fig. 3:**
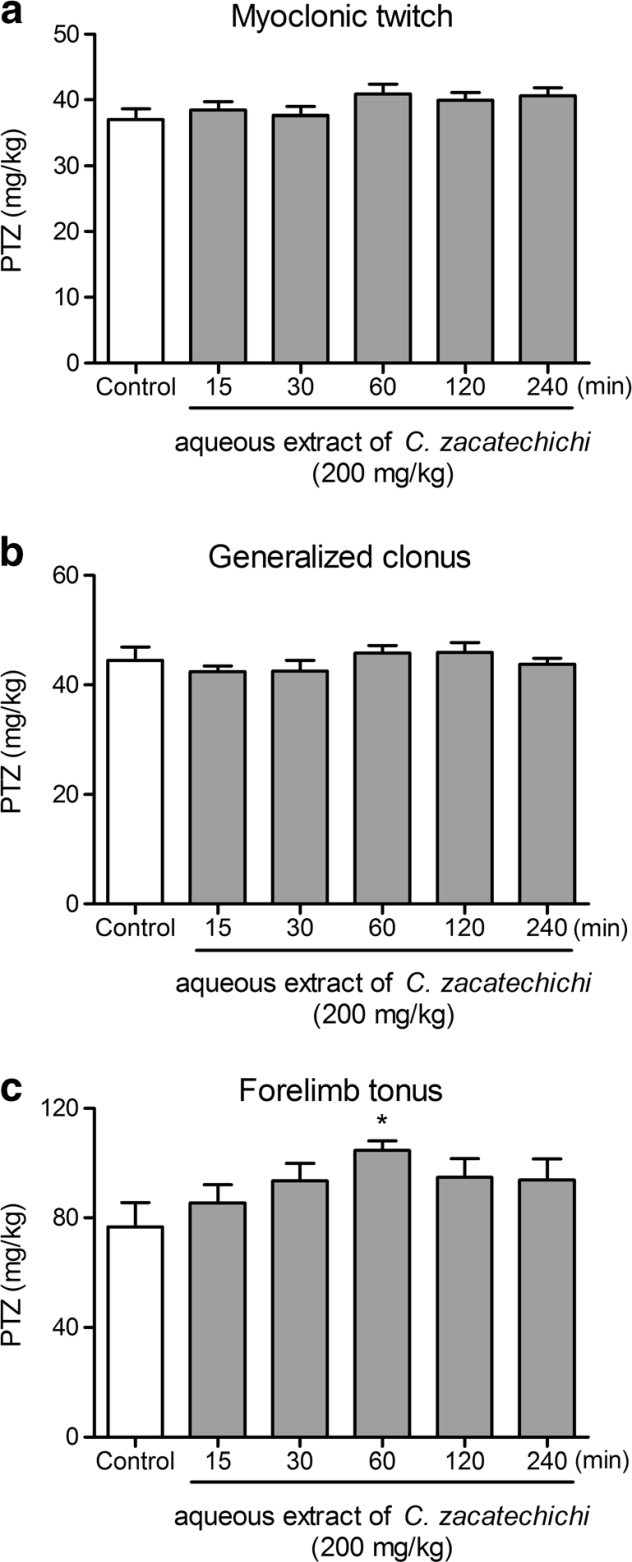
The time course of the changes in pentylenetetrazole seizure threshold after a single administration of the aqueous extract of *C. zacatechichi* (200 mg/kg, p.o.). Figure shows data for myoclonic twitch (**a**), generalized clonus (**b**) and forelimb tonus (**c**) which is significantly increased 60 min after administration. Data represent mean + SEM of *n* = 10–14 mice for each experimental group. *P < 0.05, as compared to control

We also investigated the influence of the aqueous extract of *C. zacatechichi* on PTZ-induced seizure threshold. The aqueous extract of *C. zacatechichi* administered p.o. at the doses ranging from 200 to 800 mg/kg did not alter any of the measured parameters, including myoclonic twitch (Fig. 2a), onset generalized clonus (Fig. 2b), and tonic forelimb extension (Fig. 2c). Moreover, we investigated the time course of the changes in measured parameters after a single administration of the extract (200 mg/kg, p.o., Fig. 3). Only the tonic forelimb extension was significantly affected by the extract with the maximum effect observed after 60 min post administration (Fig. 3c).

### Aqueous extract of *C. zacatechichi* produces depressive-like effects in the forced swim test

**Fig. 4:**
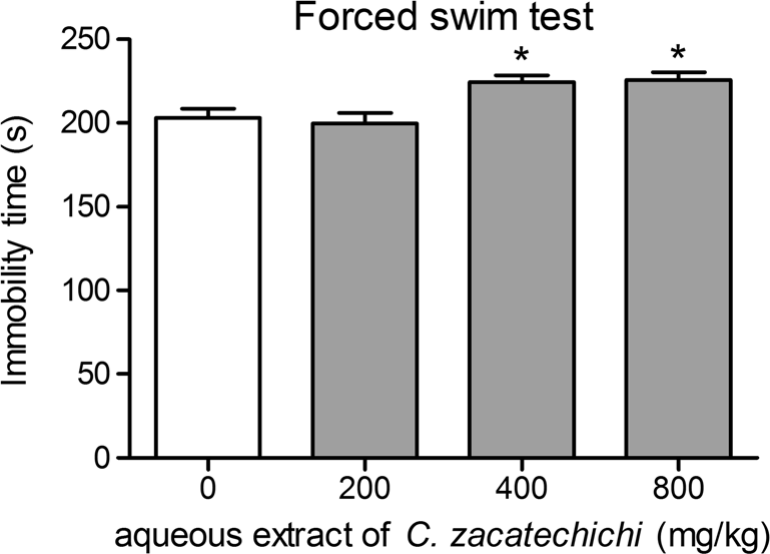
Effect of the aqueous extract of *C. zacatechichi* (200–800 mg/kg, p.o.) on the antidepressant-like behavior in the forced swim test. Figure shows data for immobility time. Data represent mean + SEM of *n* = 10–12 mice for each experimental group. *P < 0.05 as compared to control

In order to investigate the antidepressant-like effect of the aqueous extract of *C. zacatechichi*, we performed a forced swim test in mice. As shown in Fig. 4, the p.o. administration of the aqueous extract of *C. zacatechichi* at doses of 400 and 800, but not 200 mg/kg significantly prolonged the immobility time of mice.

### Aqueous extract of *C. zacatechichi* affects neither exploratory behavior, nor anxiety levels in mice

**Fig. 5:**
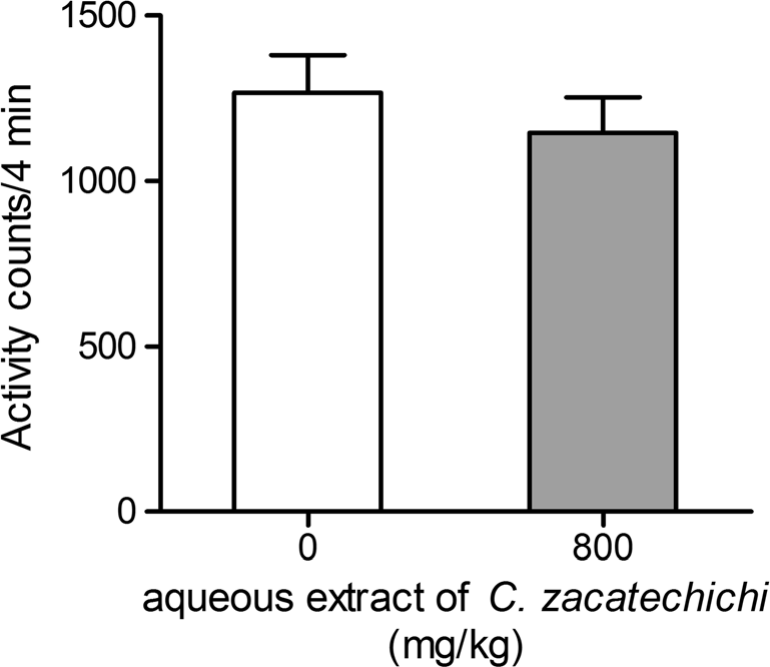
Effect of the aqueous extract of *C. zacatechichi* (800 mg/kg, p.o.) on the locomotor activity of mice. Data represent mean + SEM of *n* = 11 mice for each experimental group

The influence of the p.o. administration of the aqueous extract of *C. zacatechichi* on mouse locomotor activity was measured over a 6 min period. The extract administered at a dose of 800 mg/kg did not modify the horizontal activity (Fig. 5).

**Fig. 6:**
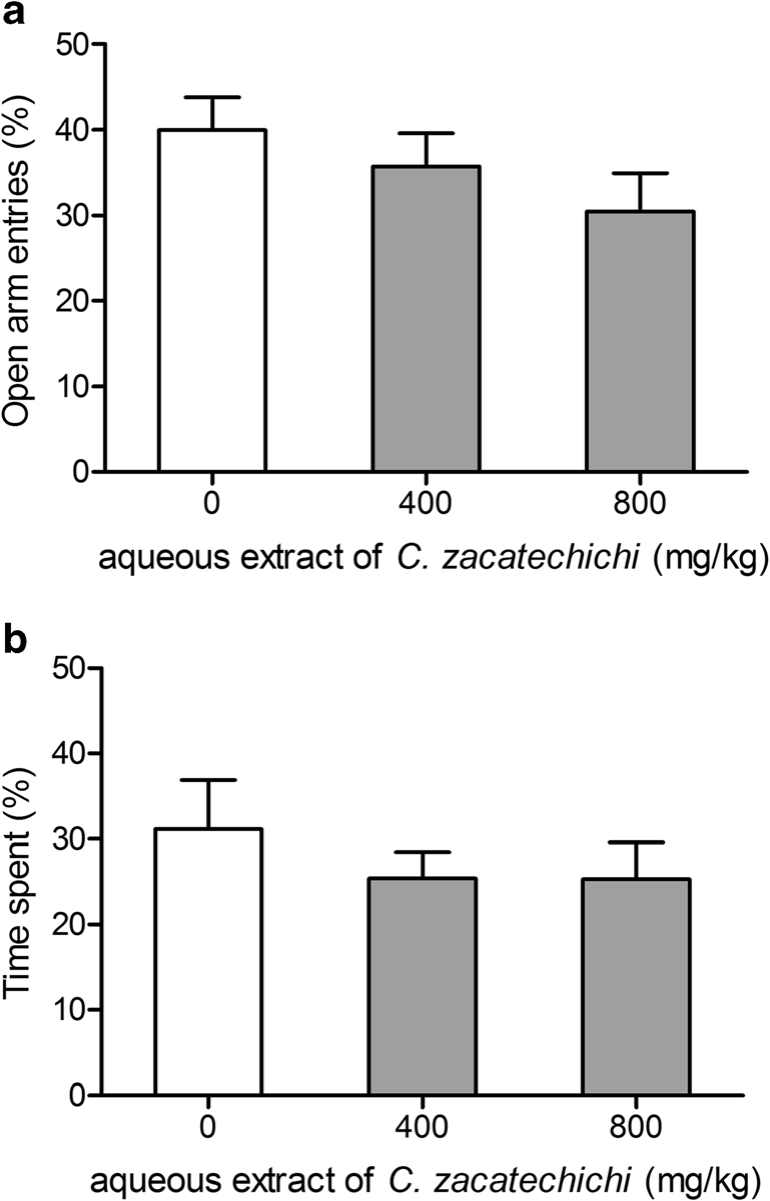
Effect of the aqueous extract of *C. zacatechichi* (400 and 800 mg/kg, p.o.) on percentage of entries (**a**) and time spent (**b**) in the opened area of elevated plus-maze. Data represent mean + SEM of *n* = 12 mice for each experimental group

To identify potential differences in exploratory behavior and changes in anxiogenic or anxiolytic activity after administration of aqueous extract of *C. zacatechichi* vs. control mice, an elevated plus-maze test was used. No significant differences were found for measures of anxiety, such as the percentage of entries (Fig. 6a) and time spent (Fig. 6b) in the open sector after p.o. administration of the extract at the dose of 400 and 800 mg/kg.

### Aqueous extract of *C. zacatechichi* does not affect muscle strength in mice

**Fig. 7:**
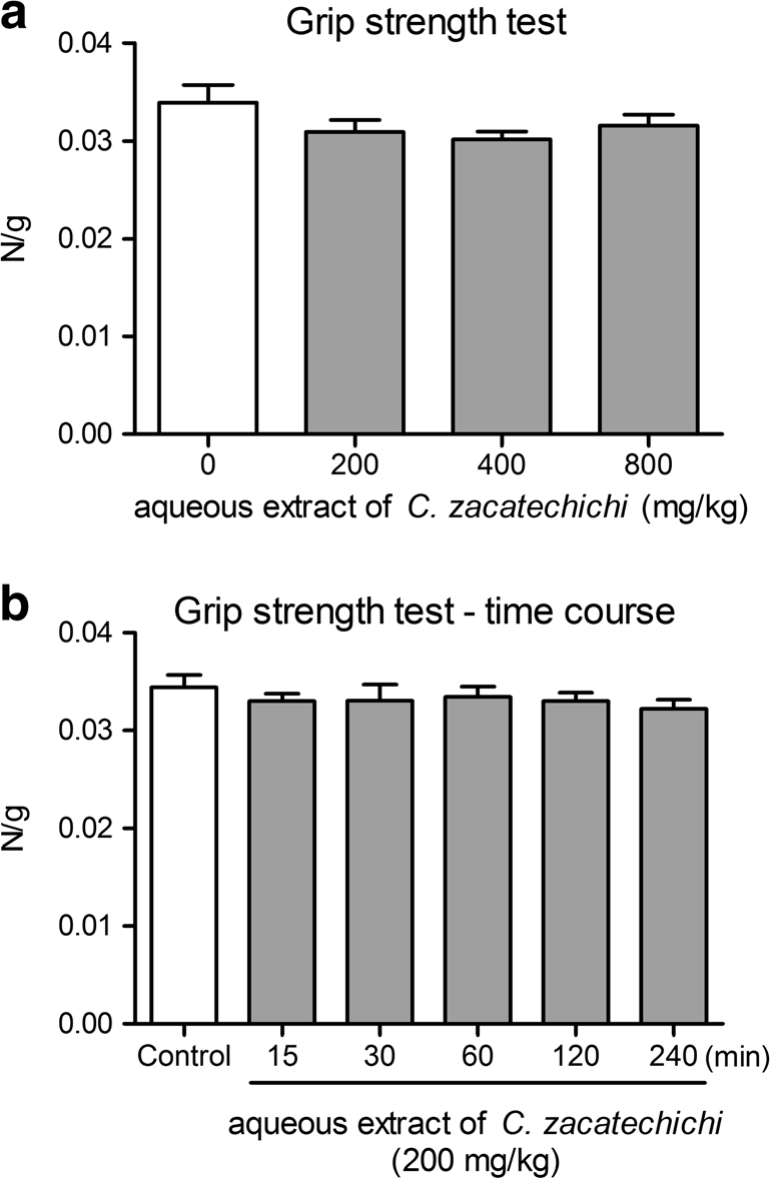
The influence of the aqueous extract of *C. zacatechichi* on muscular strength of mice. Figure shows data for the effects on muscular strength after administration at the doses ranging from 200 to 800 mg/kg, p.o. (**a**) and the time course of the changes in muscular strength after a single administration of the extract (200 mg/kg, p.o. (**b**). Data represent mean + SEM of *n* = 12 mice for each experimental group

In order to determine the effect of aqueous extract of *C. zacatechichi* on neuromotor functions of the muscles, we used grip-strength test. The extract administered at the doses ranging from 200 to 800 mg/kg, (p.o.) did not affect the grip strength in mice (Fig. 7a).

We also investigated the time course of the changes in grip strength after a single administration of the extract (200 mg/kg, p.o.). There was no effect on muscle strength between control and any of the tested time points (Fig. 7b).

### Aqueous extract of *C. zacatechichi* does not produce CNS-mediated analgesia in mice

**Fig. 8:**
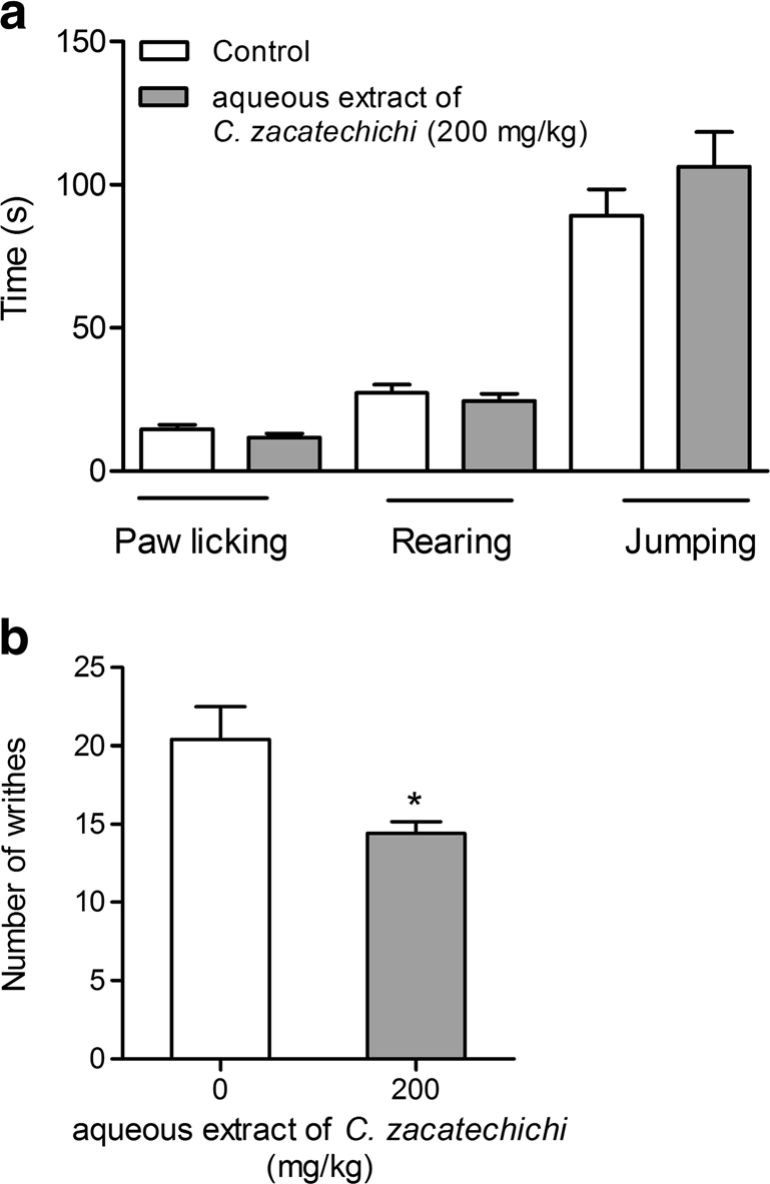
The antinociceptive effect of aqueous extract of *C. zacatechichi* (200 mg/kg, p.o.). Figure shows data for latencies of paw licking, rearing and escape jumping in the hot-plate test (**a**) and number of writhes in the writhing test (**b**). Data represent mean + SEM of *n* = 12 mice for each experimental group. *P < 0.05 as compared to control

To determine whether administration of aqueous extract of *C. zacatechichi* induces CNS-mediated antinociception we used hot-plate test. The extract administered at the dose of 200 mg/kg (p.o.) did not affect the latencies to paw licking, rearing and escape jumping (Fig. 8a).

### Aqueous extract of *C. zacatechichi* reduces visceral pain responses in mice

In order to assess the antinociceptive activity of aqueous extract of *C. zacatechichi* in the periphery we used writhing test. The p.o. administration of *C. zacatechichi* aqueous extract at the dose of 200 mg/kg resulted in a significant reduction of the number of writhes (Fig. 8b).

### Chemical composition of aqueous extract of *C. zacatechichi*

The LC-MS analysis of aqueous extract of *C. zacatechichi*, together with a high mass and high isotopic abundance match allowed us to obtain the most probable chemical formulas for all detected compounds. As shown in Table 1, the analysis revealed the presence of 6 constituents, which are quinic acid derivative (**1**), flavonol derivatives (**2, 3**), flavone derivative (**4**) and germacranolides (**5, 6**); the measured accurate ion mass [M + H]^+^ of the analyzed compounds are also reported.

**Table 1:** Chemical characterization of the *C. zacatechichi* DCM extract

No.	^a^[M + H]^+^ (*m*/*z*)	Empirical formula	Name	Reference
1	^a^355.102	C_16_H_18_O_9_	Chlorogenic acid	Zatorski et al. 2015
2	^a^611.161	C_27_H_30_O_16_	Rutin	Reference standard
3	^a^595.166	C_27_H_30_O_15_	Rutin without hydroxyl group	Reference standard
4	^a^285.076	C_16_H_12_O_5_	Acacetin	Liu et al. 2012
5	^a^407.170	C_21_H_26_O_8_	Calealactone C	Wu et al. 2011
6	^a^421.185	C_22_H_28_O_8_	Calein A	Wu et al. 2011

Alignment of obtained mass spectra with METLIN library or published papers (Chouchi and Barth 1994; Liu et al. 2012; Wu et al. 2011; Zatorski et al. 2015) resulted in the identification of following compounds: chlorogenic acid (**1**), rutin (**2**) and rutin without hydroxyl group (**3**), acacetin (**4**), calealactone C (**5**) and calein A (**6**).

## Discussion

In this study we characterized the neuropharmacological and antinociceptive effects of the aqueous extract of *C. zacatechichi* in vivo. We evidenced that oral administration of the extract does not affect most of tested neurological and behavioral parameters, namely MEST, 6 Hz-induced seizure, PTZ-induced seizures, locomotor activity, plus-maze test measurements, grip strength and CNS-mediated antinociception. However, we observed significant action in the forced swim test and on one of the PTZ-induced seizures parameters, as well as significant antinociceptive effect in the model of abdominal pain.

There are several scientific reports showing beneficial actions of extracts obtained from *C. zacatechichi*, including anti-microbial, anti-inflammatory and anti-diarrheal effects (Bork et al. 1997; Wu et al. 2011). It has been suggested that the potential therapeutic use of *C. zacatechichi* may be hampered by adverse CNS-related events and there are already some evidences in the literature for its potential psychopharmacological effects. However, these data are poorly characterized. For example, Mayagoitia et al. have shown that *C. zacatechichi* extracts increase the superficial stages of sleep and the number of spontaneous awakenings in humans (Mayagoitia et al. 1986). It was also reported that volunteers subjected to the *C. zacatechichi* extracts experienced more intense dreams during the sleep (Mayagoitia et al. 1986). Here we provide novel, relevant data, which extend the existing knowledge and show the possible negative effects of the compounds occurring in the aqueous extract of *C. zacatechichi*.

To begin with, we used three seizure models, which are broadly applied to test novel anti-convulsant drugs; namely MEST, 6 Hz-induced seizure and PTZ-induced seizures. The aqueous extract of *C. zacatechichi* did not change any of the measured parameters, except for a slight increase in the dose of PTZ necessary to induce forelimb tonus 60 min after administration of the extract. Of note, this effect was not dose-dependent and was present only in the time course experiment. These observations may be perceived as a virtue of the tested extract, as many hazardous, psychoactive and/or addictive compounds are known to dramatically alter PTZ convulsion threshold. For instance, (Himmel 2008) has shown that diazepam (10 mg/kg, p.o.) and morphine (10 mg/kg, i.p.) significantly increased PTZ convulsive threshold, while isoniazide (120 mg/kg, s.c.) and yohimbine (8 mg/kg, s.c.) displayed significant and reliable proconvulsive effects. On the other hand, generally safe compounds, such as caffeine and acetylsalicylic acid remained ineffective in this test.

Moreover, the aqueous extract of *C. zacatechichi* did not alter the CC_50_ in the MEST test, suggesting the lack of CNS-related effects. Previously published data show that well-known psychoactive compounds, such as amphetamine and its derivatives, cocaine and opioids typically increase seizure threshold at small doses and produce convulsions at higher ones (Bankstahl et al. 2012). The measurements of locomotor activity and several parameters in the elevated plus-maze showed changes neither in exploratory, nor in anxiety-related behaviors after administration of the aqueous extract of *C. zacatechichi.* This is in line with our previous observations, showing the lack of changes in exploratory behavior after administration of dichloromethane extract of *C. zacatechichi* in mice, especially since these two extracts contained similar groups of compounds (Sałaga et al. 2015).

The aqueous extract of *C. zacatechichi* was inactive in the 6 Hz psychomotor seizure threshold test. We performed this test in a standard way, i.e., scoring motor seizure and postictal depression as total seizure response, without getting and analyzing the EEG. This might be a limitation of this and other studies that utilize standard 6 Hz seizure test procedure because without the EEG it is not possible to distinguish the convulsive reaction from the postictal immobility, especially when testing substances that have demonstrated effects on the CNS functions (Giordano et al. 2015). The duration of postictal depression may be modulated more than the convulsive phase by centrally-active substances.

Of interest, our data showed a significant increase in the immobility time induced by two doses (400 and 800 mg/kg, p.o.) of the aqueous extract of *C. zacatechichi* in a forced swim test. This observation suggests that the extract may have a depressant effect in mice tested in this paradigm. However, the effect was not dose-dependent and thus requires further investigation, preferably in other paradigms. The LC-MS analysis of the aqueous extract of *C. zacatechichi* revealed presence of three major groups of compounds: chlorogenic acid, acacetin (and most likely also its derivatives), rutin and rutin derivative as well as germacranolides.

Chlorogenic acid is a polyphenol and the ester of caffeic acid and quinic acid that can be found in coffee and black tea, with potential antioxidant and anti-inflammatory activities (Zatorski et al. 2015). In this study we observed that the aqueous extract of *C. zacatechichi*, which contains chlorogenic acid, elicited antinociceptive effect in the mouse model of abdominal pain.

This is in line with several studies demonstrating that chlorogenic acid inhibits abdominal constrictions induced by acetic acid as well as p-benzoquinone (Chen et al. 2013; Küpeli et al. 2012; Li et al. 2009). Moreover, pure chlorogenic acid reduces pain in neuropathic pain models (Bagdas et al. 2013; Bagdas et al. 2014). One of the suggested mechanisms by which chlorogenic acid alleviates pain is inhibition of acid-sensing ion channels in peripheral neurons (Qu et al. 2014). Noteworthy, chlorogenic acid does not induce antinociceptive effect in the hot-plate test. On the other hand, chlorogenic acid does not affect the CNS-related functions, such as cognitive performance and mood-related behaviour suggesting that this compound is not responsible for the depressant-like effect of the aqueous extract of *C. zacatechichi* reported in this study (Camfield et al. 2013).

Other biologically active compounds found in the extract were rutin and rutin without one hydroxyl group. Rutin is a glycoside of quercetin and rutinose occurring in buckwheat and rhubarb (Kreft et al. 1999). Rutin is a highly potent molecule due to its strong antioxidant properties. It has been shown previously that plant extracts containing high concentration of rutin as well as rutin itself induce visceral antinociception in mice (Lapa et al. 2009; Selvaraj et al. 2014). Therefore these compounds may be involved to some extent in the antinociceptive effects observed in our study. On the other hand, rutin isolated from the *Schinus molle* L. exhibits antidepressant-like effect in the tail suspension test without the effect on forced swim test and locomotor activity after oral administration (Machado et al. 2008). Furthermore, as shown by Dimpfel (2009), rutin and quercitin exhibit anti-depressant activity in rats through inhibition of monoamine oxidase and elevation of serotonin and noradrenalin availability. Based on these reports we suggest that rutin occurring in the aqueous extract of *C. zacatechichi* should not be considered as responsible for its depressant-like effect in vivo.

On the other hand, Martínez-Vázquez et al. (2012) have recently demonstrated that the aqueous extract of *Dracocephalum moldavica* L. (*Lamiaceae*) also produce a depressant-like effect in forced swim test in mice. The chemical analysis of that extract revealed the presence of acacetin and its derivatives in high concentrations, which may be responsible for observed depressant activity (Martínez-Vázquez et al. 2012). Our analysis also showed the presence of acacetin in the aqueous extract of *C. zacatechichi* thus the responsibility of this compound for the increase of immobility time in the forced swim test cannot be excluded.

The last group of compounds detected by our LC-MS analysis are germacranolides, including calealactone C and calein A. There are only a few papers characterizing potential biological activity of germacranolides, all of which show their anti-microbial and anti-oxidative activity, but none of them assessed their activity in vivo (Umemura et al. 2008; Wu et al. 2011). This opens up a novel field for further investigations characterizing the in vivo effects of isolated germacranolides.

## Conclusion

The aqueous extract of *C. zacatechichi* does not affect basic neurological functions or anxiety and exploratory behavior in mice after oral administration. On the other hand, it has been demonstrated in the literature that this plant preparations exhibit potent beneficial activities both in vitro and in vivo. Further chemical analyses of *C. zacatechichi* are thus urgently needed in order to find its all active constituents and identify which of them are responsible for the effects observed in vivo. Such findings may open up novel opportunities in design and synthesis of novel drugs.
